# Human-likeness and attribution of intentionality predict vicarious sense of agency over humanoid robot actions

**DOI:** 10.1038/s41598-022-18151-6

**Published:** 2022-08-16

**Authors:** Cecilia Roselli, Francesca Ciardo, Davide De Tommaso, Agnieszka Wykowska

**Affiliations:** Social Cognition in Human Robot Interaction, Center for Human Technologies, Italian Institute of Technology, Via Enrico Melen 83, 16152 Genova, Italy

**Keywords:** Human behaviour, Cognitive neuroscience

## Abstract

Sense of Agency (SoA) is the feeling of being in control of one’s actions and their outcomes. In a social context, people can experience a “vicarious” SoA over another human’s actions; however, it is still controversial whether the same occurs in Human–Robot Interaction (HRI). The present study aimed at understanding whether humanoid robots may elicit vicarious SoA in humans, and whether the emergence of this phenomenon depends on the attribution of intentionality towards robots. We asked adult participants to perform an Intentional Binding (IB) task alone and with the humanoid iCub robot, reporting the time of occurrence of both self- and iCub-generated actions. Before the experiment, participants’ degree of attribution of intentionality towards robots was assessed. Results showed that participants experienced vicarious SoA over iCub-generated actions. Moreover, intentionality attribution positively predicted the magnitude of vicarious SoA. In conclusion, our results highlight the importance of factors such as human-likeness and attribution of intentionality for the emergence of vicarious SoA towards robots.

## Introduction

Sense of Agency (SoA) is the feeling of being in control of one’s actions and their outcomes^1^ occurring in the external environment^2^. In the last twenty years, the Intentional Binding (IB) paradigm has been extensively used to investigate implicit SoA^3,4^, demonstrating that voluntary actions and their sensory outcomes are perceived as shifted towards each other in time. This effect has been termed the Intentional Binding (IB) effect^3^. Notably, the IB effect can emerge not only in relation to one’s actions, but also towards other humans’ actions^5^, leading to a “vicarious” SoA. Interestingly, recent evidence showed that also artificial agents could induce vicarious SoA in humans or have an impact on SoA in general^6^. For example, a recent experiment examined how SoA- measured by the IB paradigm- was influenced when participants’ actions were performed following instructions given by another human vs. a humanoid robot, as compared to when they were freely selected^7^. Although the IB effect was stronger when participants freely selected their actions among four keypress alternatives, results also showed that the more people attributed human-like characteristics to the robot, in terms of anthropomorphism, likeability, and perceived intelligence^8^, the stronger the IB effect that emerged over robot-instructed actions^7^. Interesting findings have also been reported when investigating the SoA phenomenon in virtual reality (VR) settings. For instance, when participants were placed in a VR environment when they were instructed to perform movements displayed on the screen from the first-person perspective of a human-like avatar, they experienced an increased SoA over those movements after two training sessions compared to the control group, which was not trained using the VR environment^9^. Moreover, a recent study^10^ investigated whether the IB effect was modulated by body-specific information. The authors used VR to manipulate two key aspects of movement feedback. First, the shape: participants viewed a virtual hand or a sphere. Second, the movement congruency: the viewed object moved congruently or incongruently with the participants’ hidden hand. Results showed that only movement congruency, and not the shape, influenced the IB effect^10^. Therefore, it may be plausible that, when investigating the vicarious SoA phenomenon in VR settings, the human-like (anthropomorphic) shape is not a crucial factor. Indeed, when controlling virtual avatars, SoA seems to emerge also when the avatars do not have a human-like shape, as in the case of point-line avatars^11^, when they drastically deviate from the shape of the physical body^12^, and even when virtual limbs are presented in implausible positions^13^.

On the contrary, when investigating the vicarious SoA phenomenon in real physical settings shared with embodied artificial partners, the human-like (anthropomorphic) shape seems to be an important factor. For instance, evidence in Human–Robot Interaction (HRI) suggests that an embodied anthropomorphic hand in action showed similar IB effects as other humans did^14^. However, the vicarious IB effect was not found for actions performed by a robot that did not display human-like features^15^. Therefore, comparing results from VR and physical settings, it appears that different factors might play a role in the emergence of vicarious SoA towards artificial agents.

The fact that in real physical settings the characteristics of embodiment (human-like vs. non-anthropomorphic) play a role in SoA might be related to how much the embodied action of another agent evokes sensorimotor representation of that action in the human observer. It is plausible that the more human-like features an artificial agent displays, the more accurately humans represent their actions at the sensorimotor level. Thus, in consequence, the more likely it would be to observe vicarious SoA. This reasoning is grounded in the idea that implicit SoA depends on one’s ability to form a sensorimotor representation of an action^16–19^. A recent IB study speaks in favor of this reasoning^20^. In that study, participants performed an IB task, judging the time of occurrence of actions generated by the non-anthropomorphic Cozmo robot, and the actions’ sensory consequences (auditory tones). Cozmo was programmed to perform either physical or digital actions. Results showed that vicarious SoA for robot’s sensory outcomes emerged only when the causing actions were physical, and not in the “digital” action condition. This was interpreted as resulting from the fact that digital action-outcome links generated by artificial agents might not elicit a sensorimotor representation of an action. Interestingly, in the same study, the authors observed that attribution of intentionality^21,22^ also played a role in vicarious sense of agency towards a non-anthropomorphic robot.

## Aims

Bearing in mind the importance of human-like features of an embodied agent for the emergence of SoA, the present study investigated vicarious SoA in the context of an IB paradigm performed with the humanoid robot iCub^23^. *Humanoid robot* means that it is relatively similar to a human shape, with similar effectors as humans. In addition, we examined whether vicarious SoA towards the humanoid robot depends on the attribution of intentionality towards it. Participants performed an IB task^3,5,24^ alone and with the robot. To test the potential role of the attribution of intentionality towards robots, before the experiment participants filled out the Waytz questionnaire^25^, which measures the individual level of likelihood of attribution of intentionality towards robots.

We hypothesized that, if the human-like shape of the robot and its effectors is sufficient to induce the vicarious SoA, then a comparable IB effect should emerge for both self-generated and iCub’s actions, with no relation between the IB effect and the individual likelihood of attributing intentionality to robots. In contrast, if the attribution of intentionality plays a role in vicarious SoA, one would expect that the higher degree of attributed intentionality to robots in general, the stronger the vicarious IB effect at the individual level.

## Materials and methods

### Participants

Thirty-four participants were recruited to participate in the study (age range: 18–45 years old, M _age_ = 26.5, SD _age_ = 6.14, 4 left-handed, 16 males). All participants had a normal or corrected-to-normal vision, and they were naïve to the purpose of the study. The sample size was determined based on a priori power analysis estimating the sample needed to obtain reliable results. We used the *pwr* package^26^ in R Studio v.4.0.5^27^, considering *f*^*2*^ as the most reliable effect size measured for mixed-effects models^28^, which were planned for the analyses. We used a medium-to-large effect size (*f*^*2*^ = 0.3); the significance level (alpha) was set to 0.05, and the power of the test was set to 0.95. Results showed that a sample size of N = 30 was needed to obtain reliable results. We tested 34 participants to account for the potential exclusion of some participants from analyses. The study was conducted with the approval of the Local Ethical Committee (Comitato Etico Regione Liguria) and under the ethical standards laid down in the 2013 Declaration of Helsinki. All participants gave written informed consent before the experiment, and they were all paid 15 € for their participation. After the experiment, all participants were debriefed about the purpose of the study.

### Apparatus and stimuli

**Figure 1:**
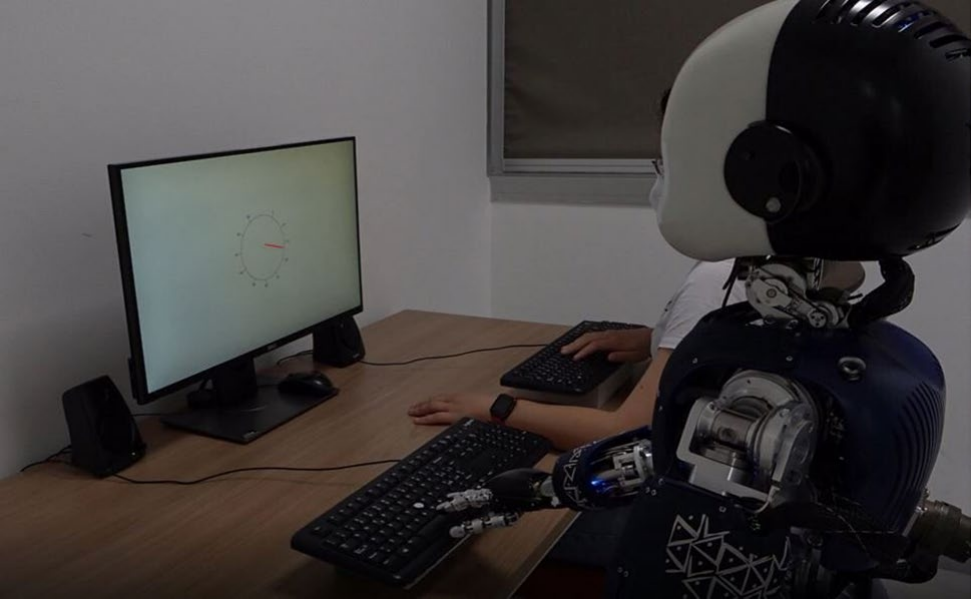
Experimental setup.

The experimental apparatus comprised the iCub robot^23^, a workstation equipped with two 27’ inches screens to display the task (resolution 1920 × 1200), two sets of speakers, and two identical QWERTY keyboards, one for participants and one for the iCub (see Fig. 1; written informed consent for publication of Fig. 1 was obtained). Participants were seated at approximately 70 cm from the computer screen. Stimuli presentation and response collection were controlled using Psychopy v2021.2.0^29^. The humanoid robot iCub^23^ was connected to the workstation using a peer-to-peer Ethernet connection (see Supplementary Materials, point SM.2, p. 4, for additional information about how the robot was integrated and controlled). The Waytz questionnaire was presented using OpenSesame v.3^30^.

### Procedure

Before the task, participants filled out the Waytz questionnaire^25^. Subsequently, they performed the IB task both alone (Solo Context) and with the iCub robot (Social Context).

Each Context (Solo, Social) included two types of (sub-) blocks of 40 trials each, i.e., a Baseline and an Operant block, presented in a randomized order. In the Baseline block, the critical event (i.e., action) did not produce any tone outcome, whereas in the Operant block the action produced a tone outcome 250 ms thereafter (440 Hz, 100 ms; see Supplementary Materials, point SM.1, p. 2, for more information). In the Solo Context, participants executed the task alone, with iCub being in a separate room. In the Social Context, participants entered the room where the robot was already activated, with its hand placed over its keyboard. In the Solo Context, participants’ task was to perform a keypress at the time of their choosing, and subsequently report the time at which the keypress was made. In the Social Context, the task was to report the time at which iCub performed a keypress. A practice session (i.e., sixteen trials, four for each combination of Block and Context) was administered before the task.

### Trial sequence

At the beginning of each trial, a fixation dot appeared on the screen for 1000 ms, followed by the image of a clock with a red clock hand (length = 135 pixels) in a static position for 500 ms. Afterward, the clock hand started rotating clockwise, with each rotation lasting 2560 ms. For each trial, the maximum number of rotations was set to 10. The clock hand stopped rotating randomly between 1500 and 2500 ms after the action occurred. In the Solo Context, participants were instructed to wait until the end of the first full rotation of the clock hand, and then to perform a keypress at the time of their choosing. In the Social Context, the iCub robot was programmed to perform a keypress at a random time after the first full rotation of the clock hand, within a predefined time window (2500–8000 ms).

At the end of each trial, participants’ task was to report the time indicated by the clock hand when they—or iCub—performed the keypress. To make sure that participants were attending iCub’s actions, the robot was programmed to press in 90% of trials. Participants were instructed that if iCub did not act before the end of the tenth rotation, they had to execute the keypress themselves; otherwise, they would lose 10 points from a starting amount of 120 points.

## Vicarious sense of agency

### Statistical analyses

For each trial, we estimated the Judgment Error (JE), namely the “minute” difference between the position of the clock hand on the clock display reported by participants and its actual position when the keypress occurred. Then, “minute” JEs were transformed into “millisecond” JEs (minute JEs × 2560 ms/60). For each Block type (Baseline, Operant) we calculated the mean JEs and their standard deviations. JEs that deviated more than ± 2.5 SD from the participants’ mean for each type of Block were considered outliers and removed from the analyses (3.38% of the total number of trials; mean JEs = 26.9 ms, SD = 435.77 ms). Data of three participants were excluded due to a low number of remaining trials in the Social Context after outliers’ removal (< 30 trials in Baseline or Operant block, or both), resulting in a sample size of N = 31. Then, JEs were modeled as a function of Block type (Baseline, Operant) and Context (Solo, Social), plus their interactions, as fixed effects and participant as a random effect. Note that the IB for action events, namely the *Action Binding* effect, is defined as less negative JEs of the time of the action event for the Operant block, relative to the Baseline block^20^. Analyses were conducted using the *lme4* package^31^ for linear mixed-effects models in R studio v. 4.0.5^27^. Parameters estimated (β) and their associated t-tests (t, p-value) were calculated using the Satterthwaite approximation method for degrees of freedom^32^; they were reported with the corresponding bootstrapped 95% confidence intervals^33^.

### Results

**Figure 2:**
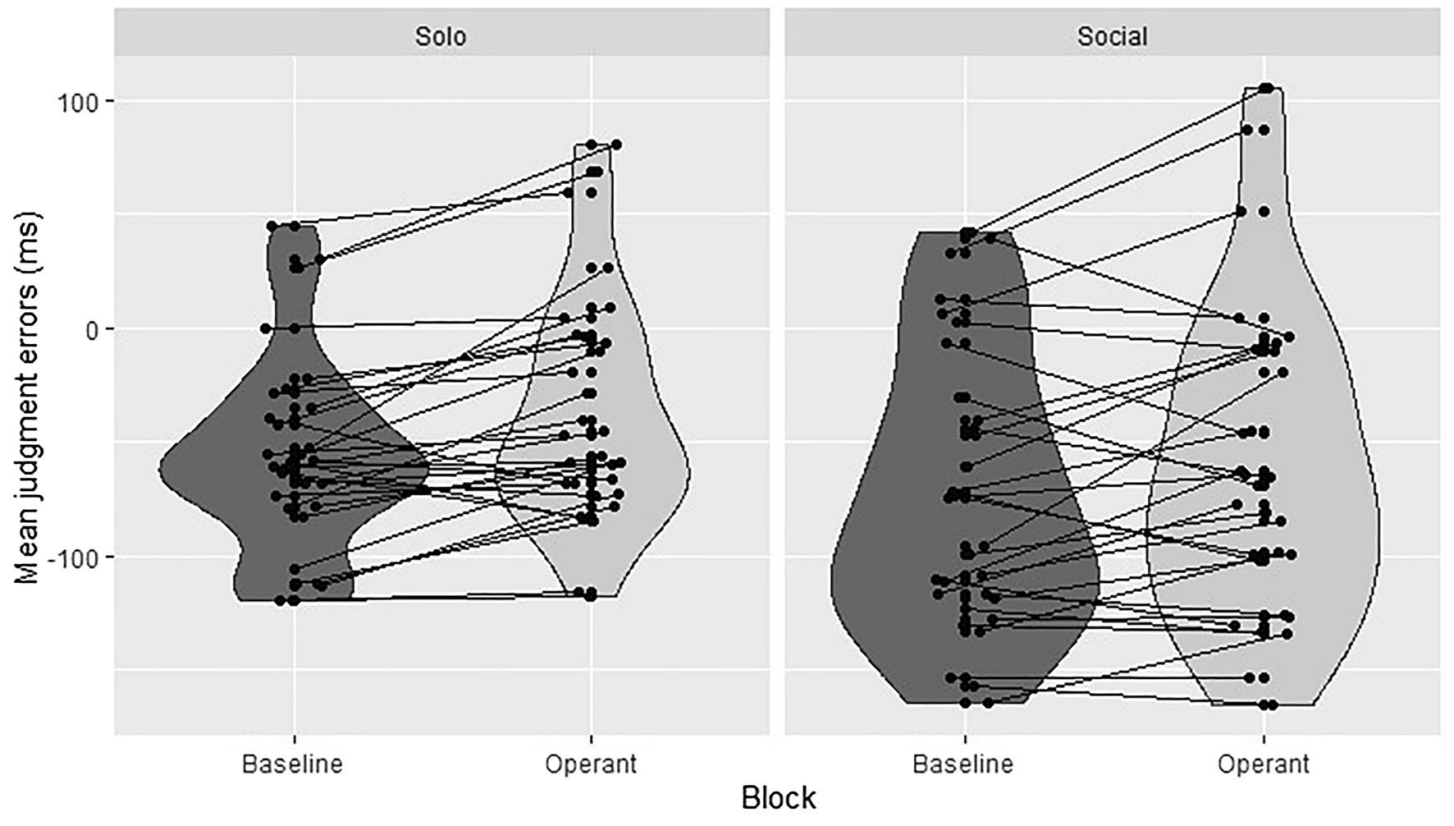
Mean JEs plotted as a function of Block (Baseline, Operant), separately for each Context (Solo, Social). Data points are plotted in pairs to illustrate the effects at the individual level.

Results showed a significant main effect of Block type [β = 10.56, SE = 3.13, t_(30)_ = 3.37, p = 0.0007, CI (4.42; 16.69)], with less negative JEs in Operant compared to Baseline blocks [β = − 14.7, SE = 2.16, t_(30)_ =  − 6.81, p < 0.0001, CI (− 18.9; − 10.5); (M_Operant_ = − 49.9 ms, SE_Operant_ = 8.74; M_Baseline_ = − 64.6 ms, SE_Baseline_ = 8.74)]. Moreover, a significant main effect of Context emerged [β = 16.01, SE = 3.04, t_(30)_ = 5.25, p < 0.0001, CI (10.04; 21.99)], with less negative JEs in Solo compared to Social Context [β = − 20.1, SE = 2.16, t_(30)_ =  − 9.34, p < 0.0001, CI (− 24.4; − 15.9); (M_Solo_ = − 47.2 ms, SE_Solo_ = 8.74; M_Social_ = − 67.3 ms, SE _Social_ = 8.74)]. Notably, the two-way Block * Context interaction was not significant [β = 8.24, SE = 4.31, t_(30)_ = 1.91, p = 0.05, CI = (− 0.2; 16.69)] (see Fig. 2).

## Intentionality attribution

### Statistical analyses

To test the relationship between attribution of intentionality, as indexed by the Waytz score^25,34^ and the vicarious SoA, we selected only trials in the Social Context, i.e., when participants reported the time of occurrence of iCub’s actions. Data of another participant were excluded due to unaccomplished completion of the Waytz questionnaire, resulting in a sample size of N = 30 for this analysis. Social JEs were modeled as a function of both Block type (Baseline, Operant) and Waytz score, plus their interactions, as fixed effects, and participant as a random effect. Analyses were conducted using the *lme4* package^31^ in R studio v. 4.0.5^27^. Parameters estimated (β) and their associated t-tests (t, p-value) were calculated using the Satterthwaite approximation method for degrees of freedom^32^, and then reported with the corresponding bootstrapped 95% confidence intervals^33^.

### Results

**Figure 3:**
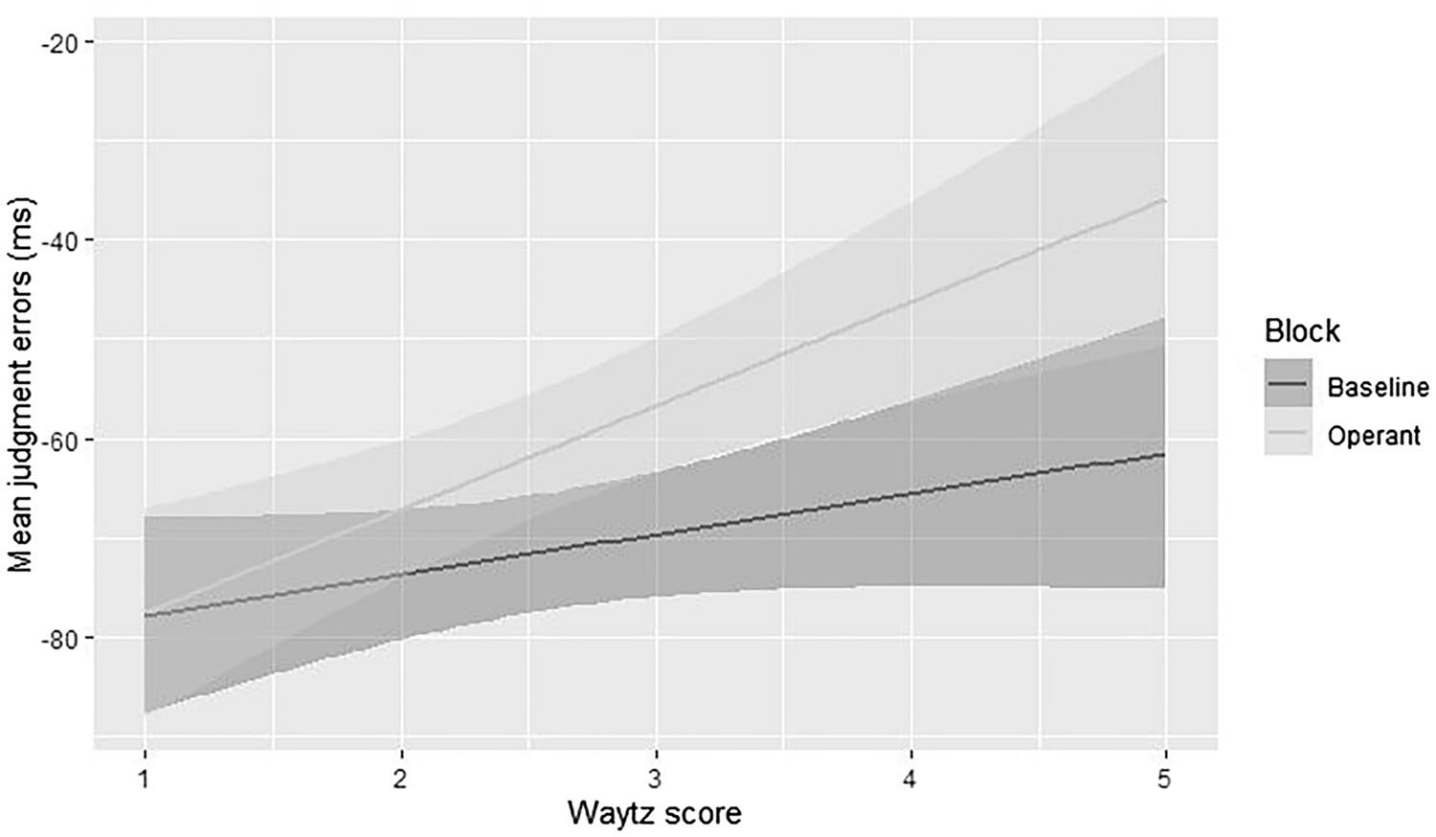
Mean JEs in the Social Context plotted as a function of Waytz score for both Baseline and Operant block.

Results showed no main effect of Block [β = − 5.85, SE = 8.52, t_(29)_ =  − 0.68, p = 0.49, CI = (− 22.55; 10.84)]. Moreover, no significant main effect of Waytz emerged [β = 4.34, SE = 10.09, t_(29)_ = 0.43, p = 0.67, CI = (− 15.38; 24.07)]. Notably, the two-way Block * Waytz interaction resulted to be significant [β = 6.4, t_(29)_ = 2.1, p = 0.03, CI (0.44; 12.35)]. Specifically, Waytz score predicted JEs only in the Operant block [β = 10.41, SE = 2.78, t_(29)_ = 3.74, p = 0.0009, CI (4.96; 15.87)], and not in the Baseline block [β = 4.07, SE = 2.58, t_(29)_ = 1.57, p = 0.11, CI (− 0.99; 9.13)] (see Fig. 3).

## General discussion

The present study examined whether humanoid robots can elicit vicarious SoA, and whether it is related to the attribution of intentionality towards robots. To address the aims of the study, we employed the Intentional Binding (IB) paradigm^3,5,24^. It represents a well-established measure to investigate implicit SoA^2,3^, which is computed in the form of the IB effect. Notably, it has been largely employed to shed light on how SoA emerges not only at the individual level [see^4^ for a review], but also in shared social contexts with various kinds of agents, such as other humans^17,35,36^, or artificial agents such as computers^24,35^ and robots^6,14^.

In our study, participants performed an IB task^3,5,24^ both alone (Solo Context) and with the humanoid robot iCub (Social Context). To assess the role of attribution of intentionality, participants filled out the Waytz questionnaire^25^ before the experiment. Our dependent measure was the Judgment Error (JE) in the IB task, i.e., the difference between the perceived and the actual position of the clock hand when the critical event (action) occurred.

Results showed that participants experienced SoA over both self-generated and iCub’s actions, as demonstrated by the significant Action IB effect emerging in both Solo and Social contexts. Moreover, the magnitude of SoA, in the form of the IB effect, was comparable across contexts (Solo vs. Social), as the Block*Context interaction resulted not to be significant (see Supplementary Materials, point SM.4, p. 7, for more information).

Overall, our results demonstrate that vicarious SoA emerged in relation to the humanoid robot iCub, presumably due to the robot’s human-like shape, which allowed participants to create a sensorimotor representation of its actions. Indeed, the “motor” model of SoA, namely the *Comparator Model*^37,38^, suggests that humans represent- at the sensorimotor level- the causal link between actions and their sensory outcomes. The possibility to create a sensorimotor representation of the action-outcome link would be strictly connected to the experience of agency^39^. In line with this, also vicarious SoA may rely on the same predictive mechanisms^17^. This may explain previous evidence showing a lack of vicarious SoA for robot’s digital actions or physical actions executed with a non-human effector^20^. In both cases, the action of the robot could not have allowed for an accurate sensorimotor representation^20^. Conversely, vicarious SoA emerged, in the form of the IB effect, when participants judged the occurrence of actions generated by a robotic arm resembling a human-like shape: in this case, the human-like shape might have allowed to form an accurate sensorimotor representation of the robot’s actions^14^.

In a pivotal study, Wohlschläger and colleagues already suggested the role of sensorimotor representation for the emergence of vicarious SoA, when investigating the awareness of actions performed by oneself, another human agent, or a machine^35^. The authors found that the perceived onset time of one’s actions was comparable to the perceived onset time of another human agent’s actions. However, both were substantially later than the perceived onset of a physically comparable machine action^35^_._ It resulted in more negative JEs for the machine action event compared to judgments of one’s own or other humans’ action events, suggesting a more accurate awareness of human action^2,4,40^. According to the authors, one explanation might be that the perception of the biological motion is more accurate compared to the one performed by a mechanical agent^35,41^. Notably, the same pattern as in Wohlschläger and colleagues’ study^35^ emerged in our study, as indicated by less negative JEs (i.e., closer to 0) in Solo compared to Social Context.

Interestingly, the authors of the same paper suggested another explanation, according to which vicarious SoA may relate to the degree of intentionality attributed to the co-agent^35^. They proposed that people might attribute intentions to others as well as they do to themselves, and thus the estimates of self-generated actions are comparable to the ones of the other human-generated actions; the same might not occur with machines^35^. However, further studies demonstrated that, in some contexts, people attribute intentions to machines such as robots^21,22^. In line with this, our study results showed that the magnitude of vicarious SoA was positively correlated with the degree of attributed intentionality. Specifically, the Waytz score resulted to be predictive only of JEs in Operant block, i.e., when both events (actions and tone) were present, suggesting that attribution of intentionality led participants to perceive iCub’s actions as linked to the subsequent outcome. In other words, attribution of intentionality may act as a reinforcement of the action-outcome link, and thus boost the magnitude of vicarious SoA towards robots’ actions.

Notably, our results replicate previous evidence showing a similar positive relationship between the degree of intentionality attribution and the magnitude of vicarious SoA that people experience towards actions performed by a non-anthropomorphic robot^20^. Moreover, the role of intentionality attribution towards robots in the emergence of vicarious SoA is supported by studies showing that a robot perceived as an intentional agent can affect one’s SoA, in contrast to a non-agentic, passive device^6^. In a similar vein, these studies may explain why people do not experience vicarious SoA towards artificial systems that are not perceived as intentional^16,35^. Therefore, it is plausible that vicarious SoA can be used as an implicit measure of attribution of intentionality.

Finally, it is important to mention also some limitations of the present study. One is the behavioral nature of the task. Our choice of using only behavioral measures was motivated by a vast amount of literature that used behavioral measures in the IB paradigm [see^4^ for a review]. However, in future research, it would be important to examine the neural markers of vicarious SoA towards artificial agents, as the evidence provided by our study is limited to the subjective judgments of temporal occurrence of events.

Furthermore, the nature of the task may lack ecological validity. Indeed, the IB task, which consists of reporting the position of the clock hand at the occurrence of a critical event, is not a common task in everyday life, especially if we imagine tasks that are shared with robots. Thus, future studies may consider carrying out experiments on vicarious SoA in setups of higher ecological validity.

## Conclusions

Taken together, our results demonstrate that the vicarious SoA can emerge towards a robot when it has a human-like shape. The morphological similarity between a humanoid robot and a human may allow people to generate a sensorimotor representation of the robot’s actions, similar to one’s actions. However, interestingly, the individual tendency of attributing intentionality to robots additionally “boosts” the vicarious SoA towards humanoid robots. This means that vicarious SoA might serve as an indirect implicit measure of attributed intentionality towards artificial agents.

## Supplementary Information

Supplementary Information.

## Data Availability

Datasets used for the analyses, Supplementary Materials file, and a video of the experiment can be found at the following link: https://osf.io/xj3cq/?view_only=0f4d1bdf17a6424fbc83d8f4a48011b6 (OSF repository name: “Human-likeness and attribution of intentionality predict vicarious sense of agency over humanoid robot actions). In case of acceptance of the paper, the repository will be made publicly accessible.
