# Supplementary material for: Human-likeness and attribution of intentionality predict vicarious sense of agency over humanoid robot actions
Source: Sci Rep. 2022 Aug 16;12:13845. doi: 10.1038/s41598-022-18151-6 (PMC9381554; doi:10.1038/s41598-022-18151-6)
Supplement: Supplementary file 1 — Supplementary Information. [file 41598_2022_18151_MOESM1_ESM.pdf]

Supplementary materials for

Human-likeness and attribution of intentionality predict vicarious sense of agency over humanoid robot actions

Cecilia Roselli[1], Francesca Ciardo[1], Davide De Tommaso[1], and Agnieszka Wykowska[1]

[1] Social Cognition in Human Robot Interaction, Fondazione Istituto Italiano di Tecnologia, Center for Human Technologies, via Enrico Melen 83, Genova, Italy

This file includes:

SM.1. Latency measurement

SM.2. Robot integration

SM.3. Assumptions of Linear Mixed-Effects Models

SM.3.1. Model 1: JEs ~ Block * Context + (1| Participants)

SM.3.2. Model 1: Social JEs ~ Block * Waytz + (1| Participants)

SM.4. Bayes factor for the interaction term

References

SM.1. Latency measurement

Aim. The aim was to determine whether the onset of the auditory tone outcome, as signaled by the PTB library in Psychopy v.2021.2.0 [1], matched the actual physical event produced by the audio speakers. This way, we wanted to ensure that the action-tone interval was set to 250 ms as in the classical IB paradigm based on the Libet clock method [2]. In order to obtain this latency, we measured, over many trials, time intervals between when the tone was supposed to start playing (in the code) and the onset (i.e., the start of rising edge) of the tone signal recorded by a dynamic microphone.

Equipment. Our target system subject to measure consists of the pc running code of the experiment (i.e., a workstation equipped with a 27' inches display, resolution 1920x1200) and a set of audio speakers. Our measuring system was a BioSemi EEG system used for collecting two input signals. The first one is the analog signal from a dynamic microphone placed in front of the audio speakers. The second one is a TTL signal from a Brain Product Trigger Box connected to the pc through a USB port.

Procedure. The two input signals are recorded for the entire duration of the test using the BioSemi EEG system and later analyzed as time series using MATLAB (MATLAB version R2021b) [3]. The first input is the audio signal that contains information about the actual onset/offset of the auditory stimuli played during the experiment. The second input is a TTL signal generated inside the code for triggering the event where the auditory tone was supposed to start (onset) and end (offset). The onset and offset of the auditory tone were marked with triggers S100 and S200, respectively. The test was performed both in the Solo (i.e., when participants performed the task alone) and in the Social Context (i.e., when participants performed the task with the iCub robot). Then, the signal related to the auditory tone was imported in EEGLab v.2020.0 [4]. All channels

were removed but ERGO1, i.e., the microphone channel. We also added a second channel, i.e., MIC-RECT, obtained by applying (1) a Z-transformation (subtracting the mean and dividing by the SD of ERGO1), and (2) a signal rectification, (calculating the absolute value of ERGO1).

The subsequent step was to identify the time points in which the auditory tone on the MIC-RECT channel overcame its background noise, i.e., *significant activations*. To this aim, with the signal digitized at a 2048 Hz sampling rate, as a baseline we selected a period of 1000 ms, i.e., from 0 to 1000 ms after the recording of the auditory tone started. Then, we calculated Mean and SD of the MIC-RECT signal in this 1000 ms period.

Notably, time points that deviated more than Mean ± 3 SD were considered as *significant activations*. They were observed in the time interval between S100 and S200 triggers, i.e., between the onset and the offset of the auditory tone. *Significant activations* occurring outside the S100-S200 time interval were not considered for further analyses. Then, for each couple of S100-S200 time intervals, we considered the latency of both S100 and S200 triggers. Specifically, we considered all the latencies (1) having *significant activations* (vt), and (2) occurring in this time interval. Then, we considered the minimum latency as the start of the rising edge (tmin = min(vt)), and then the difference (dt = t100 – tmin) representing the processed latency, for both the Solo and the Social Context.

Notably, in raw data we found 1 outlier due to spurious *significant activations*. Thus, we applied a 20 ms threshold on the differences to exclude these outliers and to obtain more reliable estimations.

Results. In the Solo Context, i.e., when participants performed the task alone, results showed that the actual onset of the auditory tone was slightly delayed compared to the command to play the tone [M = -36.47 ms; SD = 0.28; 95% CI $_{\text{Mean}}$ = (-37.11; -36.13)]. Similar results were found in

the Social Context, i.e., when participants performed the task with iCub [M = -36.55 ms; SD = 0.33; 95% CI $_{Mean}$ = (-37.11; -36.13)].

Conclusions. Our results showed that, on average, there is a 36 ms delay between when the command to play the auditory tone is sent and the actual onset of the tone. Therefore, considering the little variability of the latencies, the action-tone time interval was modified accordingly in Psychopy to ensure that it corresponded to 250 ms.

SM.2. Robot integration

The iCub humanoid robot iCub [5] was connected to the experimental pc using a peer-to-peer Ethernet connection. This way, we created a network shared between the experimental pc and the iCub robot. In the experimental machine was installed all the software needed for controlling the robot, namely YARP [y] and all its basic modules. Therefore, for controlling the robot from the experimental script in PsychoPy, we used the YARP Python wrappers in Psychopy [6]. Then, we used predefined postures of the robot in joint space and the standard YARP position controller (*IPositionController*) to make the robot tapping. This way, we ensured a high accuracy on the repeatability of the same movements across trials rather than using a kinematic controller in task space.

SM.3. Assumptions of Linear Mixed-Effects Models

To check the assumptions, separately for each model, all analyses have been conducted in R Studio [7].

SM.3.1. Model 1: JEs ~ Block * Context + (1| Participant)

Linearity assumption. A crucial assumption of LMMs is a linear relationship between the explanatory variables and the response variable. However, in this model we used only categorical variables as predictors in the model, namely Block (Baseline vs. Operant) and Context (Solo vs. Social). Therefore, the linearity assumption is vacuous, as it is met by definition, and can be ignored.

Homoscedasticity assumption. To test the homoscedasticity assumption, we extracted residuals from the model, take their absolute values, and then square them. Finally, an ANOVA of the between-subjects residuals was run. As the p-value did not reach the significance level ($p = 0.07$), we concluded that the homoscedasticity assumption was met.

Normality of residuals. To estimate the normality of residuals, the most informative way is to visualize whether the standardized residuals lie with respect to normal quantiles, through a normal quantile plot (see **Fig. 1**).

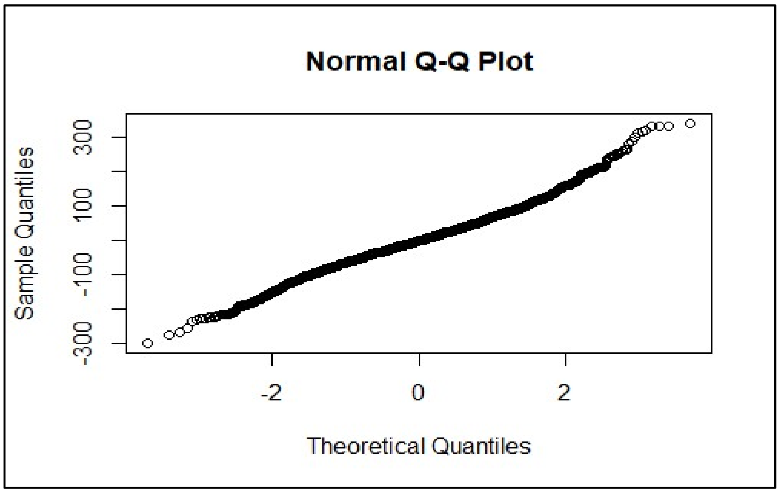

Fig. 1. Visual representation, in the form of a QQ Plot, of the normality assumption of Model 1.

As no strong deviations emerged, we concluded that the normality assumption was met. Notably, we did not use a statistical test to check the normality of the distribution (e.g., the Shapiro-Wilk test), as it is fairly useless when the model includes a huge amount of data points (5000, as a rule

of thumb) [8]. In these cases, even minute deviations would result as significantly deviating from normality, without really informing whether data are normally distributed or not.

SM.3.2. Model 2: Social JEs ~ Block * Waytz + (1| Participant)

Linearity assumption. We tested the linearity assumption by plotting the Waytz scores versus the model residuals, namely the difference between the observed values and the model-estimated value (see **Fig. 2**).

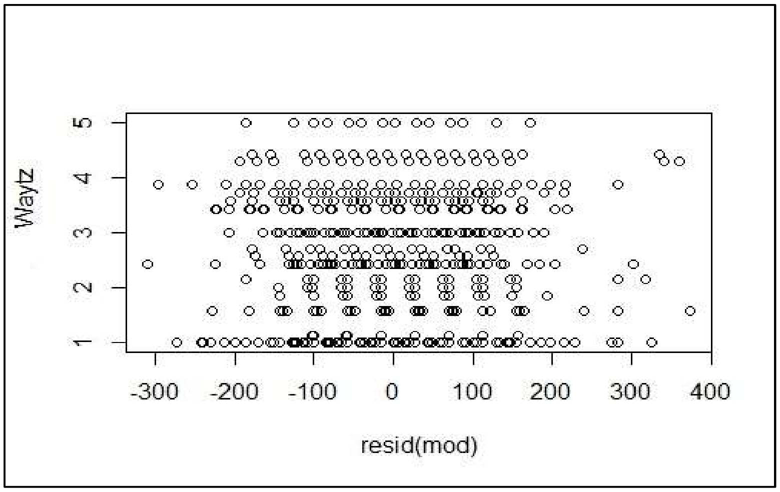

Fig. 2. Visual representation of the linearity assumption of Model 2.

As no specific patterns or trends emerged, we can conclude that the linearity assumption was met.

Homoscedasticity assumption. The residuals were transformed as described in section SM.3.1. The results showed that between-subjects residuals did not statically differ across participants (p = 0.06), suggesting that the variances of the residuals did not differ, and thus that the homoscedasticity assumption was met.

Normality of residuals. Following the same procedure described in SM.3.1, we plotted the standardized residuals to visually inspect their distribution with respect to normal quantiles (see **Fig. 3**).

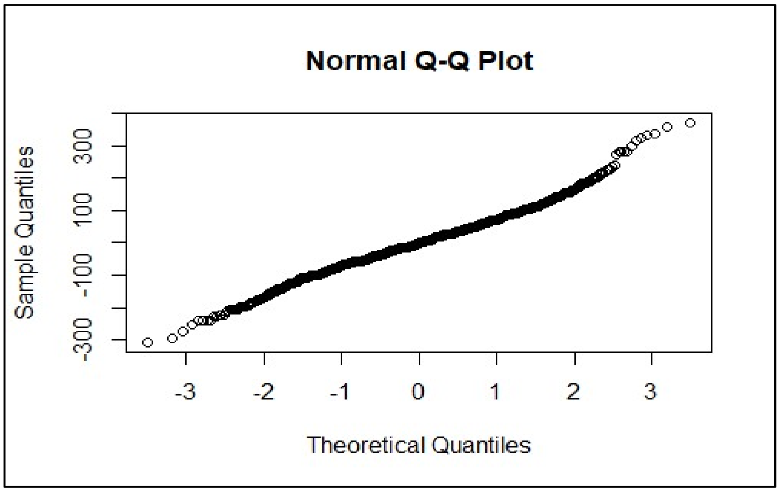

Fig. 3. Visual representation, in the form of a QQ Plot, of the normality assumption of Model 2.

As no strong deviations emerged, we then concluded that the normality assumption was met.

SM.4. Bayes factor for the interaction term

In order to further investigate the lack of the two-way Block * Context interaction reported in the manuscript (point 4.1), we calculated a BIC-based Bayes factor for the interaction term. The purpose was to understand whether the slope of JEs to Block differed by Context, while using the "Participants" random effect to discard between-subjects offsets. To this aim, we first computed a null model, which was identical to the full model we reported in our study- without the interaction term. Then, we calculated the difference between the null model and the full model, while converting BIC into the Bayes factor. Our results of the Bayesian test showed a value of 1 (0.996), which is inconclusive, meaning that our data have the same likelihood under both hypotheses [9]. Therefore, based on the Bayes factor, we do not have sufficiently strong evidence to either reject or accept the null hypothesis.

References

1. Peirce, J. W. PsychoPy—psychophysics software in Python. *J. Neurosci. Methods* **162**, 8-13 (2007). https://doi.org/10.1016/j.jneumeth.2006.11.017
2. Haggard, P., Clark, S., & Kalogeras, J. Voluntary action and conscious awareness. *Nat. Neurosci.* **5**, 382-385 (2002). https://doi.org/10.1038/nn827
3. MATLAB. version 7.10.0 (R2012b). Natick, Massachusetts: The MathWorks Inc. (2010).
4. Delorme, A., & Makeig, S. EEGLAB: an open source toolbox for analysis of single-trial EEG dynamics including independent component analysis. *J. Neurosci. Methods* **134**, 9-21 (2004). https://doi.org/10.1016/j.jneumeth.2003.10.009
5. Metta, G. et al. The iCub humanoid robot: An open-systems platform for research in cognitive development. *Neural Netw.* **23**, 1125-1134 (2010). https://doi.org/10.1016/j.neunet.2010.08.010
6. Metta, G., Fitzpatrick, P., & Natale, L. YARP: yet another robot platform. *Int. J. Adv. Robot. Syst.* **3**, 8 (2006). https://doi.org/10.5772/5761
7. R Core Team. R: A language and environment for statistical computing. R Foundation for Statistical Computing, Vienna, Austria. http://www.R-project.org/ (2013).
8. Royston, P. Approximating the Shapiro-Wilk W-Test for Non-Normality. *Stat. Comput.* **2**, 1719 (1992). https://doi.org/10.1007/BF01891203
9. Wetzels, R. et al. Statistical Evidence in Experimental Psychology: An Empirical Comparison Using 855 t Tests. *Perspect. Psychol. Sci.* **6**, 291–298 (2011). https://doi.org/10.1177/1745691611406923
